# Development of a Novel Inflammatory-Associated Gene Signature and Immune Infiltration Patterns in Intervertebral Disc Degeneration

**DOI:** 10.1155/2022/2481071

**Published:** 2022-09-22

**Authors:** Tao Lan, Zhihao Hu, Weizhuang Guo, Bin Yan, Yuantao Zhang

**Affiliations:** ^1^Department of Spine Surgery, Shenzhen Second People's Hospital, The First Affiliated Hospital of Shenzhen University, Shenzhen, Guangdong 518035, China; ^2^Musculoskeletal Research Laboratory of Department of Orthopaedics & Traumatology, Faculty of Medicine, The Chinese University of Hong Kong, Hong Kong, China

## Abstract

**Background:**

Both inflammatory factors and immune response play important roles in the pathogenesis of intervertebral disc degeneration (IDD). However, a comprehensive analysis of interaction between inflammatory response-associated genes (IRGs) and immune microenvironment in patients with IDD remains lacking. Hence, the current research is aimed at investigating the correlations between IRG signatures and immune cells in the progression of IDD.

**Methods:**

The expression profiles (GSE27494 and GSE41883) and IRGs were downloaded from the Gene Expression Omnibus (GEO) database and Molecular Signature Database (MSigDB), respectively. Weighted gene coexpression network analysis (WGCNA) and differential expression analysis were used to identify the pivotal modules and common differentially expressed genes (DEGs) associated with IDD. Subsequently, we retrieved differentially expressed IRGs (DE-IRGs) by intersecting IRGs and DEGs for enrichment analysis. Next, LASSO regression analyses were performed to screen optimal marker genes for IDD prediction. Additionally, we validated differences DE-IRGs between IDD patients and controls in GSE150408. Finally, the infiltration alteration of immune cells was evaluated by the CIBERSORT, and the correlation between diagnostic markers and infiltrating immune cells was analyzed.

**Results:**

A total of 10 upregulated differentially expressed inflammatory genes were identified that were obviously related to progression of IDD. Functional analysis results revealed that DE-IRGs were mainly enriched in signaling pathways TNF, IL-17, NOD-like receptor, and NF-kappa B pathway. A five-gene signature that consisted of IL-1*β*, LIF, LYN, NAMPT, and SLC7A2 was constructed by the LASSO Cox regression. IL1B, LYN, and NAMPT were further validated as optimal candidate genes in the pathophysiology of IDD. In addition, there was a remarkable immune cell infiltration difference between the healthy and IDD groups. The proportions for dendritic cells activated, mast cells activated, and neutrophils in the IDD group were significantly higher than those in the normal group, while the proportion of some cells was lower than that of the normal group, such as T cell CD4 memory resting, NK cells activated, and macrophage M0. Furthermore, correlation analysis indicated IL-1*β*, LYN, and NAMPT were closely implicated with immune cell infiltration in IDD development.

**Conclusions:**

We explored an association between inflammatory response-associated signature and immune infiltration in IDD and validated that IL-1*β*, LYN, and NAMPT might serve as biomarkers and therapeutic targets for IDD in the future.

## 1. Introduction

Intervertebral disc degeneration (IDD) is one of the most common degenerative diseases that is considered the leading cause of low back pain (LBP) nowadays [1, 2]. It is reported that LBP has become a prevalent medical disorder which is associated with disability and heavy socioeconomic expenditure [3]. Despite substantial progress and improvement in the management of discogenic LBP, long-lasting clinical therapy effect remains unsatisfactory. Although the etiology of IDD is multifactorial, including genetic predisposition, aging, overloading, and lifestyle (smoking and obesity), they lead to common pathological changes which is characterized by extracellular matrix degradation and excessive cell apoptosis.

Inflammation is widely accepted as an important driver of IDD progression which promotes matrix degradation, cell senescence, and apoptosis by recruiting a large number of inflammatory cytokines, such as interleukin-1*β* (IL-1*β*) and tumor necrosis factor-*α* (TNF-*α*). For example, a study conducted by Kim et al. showed that direct injection of IL-1*β* into rat lumbar intervertebral disc resulted in disc degeneration and inflammation microenvironment [4]. TNF-*α* plays a proinflammatory effect within disc and promotes nucleus pulposus cells death [5]. Conversely, anti-inflammatory treatments provide positive therapeutic effect in alleviating IDD development. Hence, downregulation of disc inflammation response is a promising strategy in the treatment of IDD.

The nucleus pulposus (NP) is the largest avascular organ which is located at the center intervertebral disc (IVD). The unique anatomical structure isolates NP from the host immune system and therefore, NP has been identified as an immune privilege organ by Naylar et al. since 1975 [6]. However, the disruption of the NP-blood barrier due to annulus fibrosus rupture leads to exposure of NP to the host and triggers numerous immune cells infiltration and immune response.

It has been demonstrated that immune cell infiltration, including Tregs and macrophages, is involved in IDD progression [7, 8]. Noteworthy, infiltrating immune cells, such as neutrophils and T cells (CD4+, CD8+), release a large number of inflammatory cytokines and aggravate inflammation response cascade. Although biological therapy targeting immune and inflammation modulation is still in its infancy, more in-depth exploration in immune-related inflammatory response sheds light on IDD treatment.

The purpose of the current study was to explore inflammatory associated biomarkers and potential therapeutic targets for the management of IDD based on bioinformatic analysis. Firstly, IDD profiles were downloaded from the Gene Expression Omnibus (GEO) database. Then, differentially expressed inflammatory genes (DE-IRGs) that were implicated with IDD progression were obtained by WGCNA and differential expression analysis, followed by enrichment analysis and LASSO regression. Next, optimal DE-IRGs were validated by independent dataset. Finally, the association between immune infiltration alteration and inflammatory biomarkers were investigated by CIBERSORT for the first time. In all, our findings might provide novel insights into the interaction between inflammatory associated genes and immune infiltration in the pathogenesis of IDD.

## 2. Materials and Methods

### 2.1. Gene Expression Data Collection

**Figure 1:**
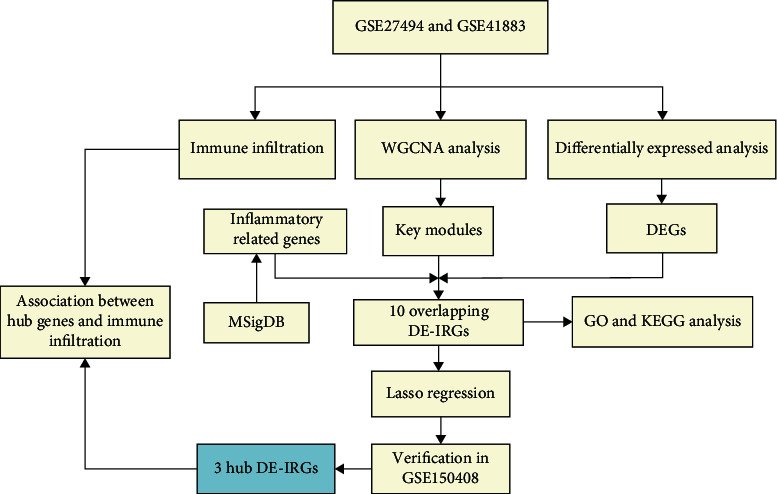
Research flow chart of this study.

The gene expression profiles of IDD, including GSE27494, GSE41883, and GSE150408 (external validation sets)), were downloaded from GEO (http://www.ncbi.nlm.nih.gov/geo) databases. The GSE27494 dataset included gene expression profiles of 4 human disc cells exposed to IL-1*β* and 4 controls. The GSE41883 dataset included gene expression profiles of 4 human disc cells exposed to TNF-*α* and 4 controls. The GSE150408 dataset included gene expression in the whole blood from 17 patients with sciatica compared with 17 healthy volunteers. The flowchart of this study was summarized in Figure 1.

### 2.2. Differential and WGCNA Expression Analyses

Differential analysis between IDD patients and healthy controls in GSE27494 or GSE41883 was performed after using the limma package in R software. A screening threshold of *P* value < 0.05 and |log_2_fold change (FC)| > 2 was set to obtain the differentially expressed genes between IDD patients and controls. Then, the heatmap and volcano plot were generated using the ggplot2 package in the R software. The WGCNA package in R was used to build a coexpression network. Then hub genes from differential analysis and key modules from WGCNA were interested to obtain DEGs. The ggpubr package was used to draw boxplot of DEGs. In addition, the correlation matrix analysis of DEGs was visualized using the different R package.

### 2.3. Functional and Pathway Enrichment Analyses

The clusterProfiler package in R was utilized to perform Gene Ontology (GO) and Kyoto Encyclopedia of Genes and Genomes (KEGG) pathway enrichment analysis of common genes. The GO analysis consisted of biological process (BP), cellular component (CC), and molecular function (MF). A *P* value less than 0.05 was considered statistically significant for enrichment.

### 2.4. Identification of Inflammatory Response-Associated DEGs

The inflammatory response-associated genes were downloaded from the hallmark gene sets in the Molecular Signature Database (MSigDB, https://www.gsea-msigdb.org/gsea/msigdb/). The IRGs overlapped with the DEGs, and then the differentially expressed IRG (DE-IRGs) genes were identified. Venn diagrams were created using the VennDiagram package.

### 2.5. Hub Genes Screening and External Validation

The least absolute shrinkage and selection operator (LASSO) method was used for the screening of the feature genes from DE-IRGs. We used the glmnet package in R to perform the LASSO logistic regression analysis. After that, selected characteristic genes were validated in an independent dataset (GSE150408), and boxplots for gene expression were drawn.

### 2.6. Immune Infiltration Related Analysis

The relative abundance of 22 human immune cells in IDD patients and healthy controls were analyzed by the CIBERSORT deconvolution algorithm, which is an important bioinformatic tool to evaluate immune infiltration microenvironment. The gene expression matrix of immune cells was downloaded from the CIBERSORT (https://cibersortx.stanford.edu) platform and matched with differentially expressed genes to generate the infiltrative proportions of immune cells in IDD. Histogram, correlation heatmap, and violin diagram were drawn to visualize the difference between the IDD and normal groups. In addition, the lollipop chart was used to analyze the relationship between immune cells and target DE-IRGs.

### 2.7. Statistical Analysis

All statistical analyses were performed using the R software. Data were shown as mean ± standard deviation (SD). The differences between the two groups were evaluated by independent sample *t*-test and nonparametric test. A *P* < 0.05 was considered statistically significant.

## 3. Results

### 3.1. Identification of 10 Differentially Expressed IRGs Related to IDD

**Figure 2:**
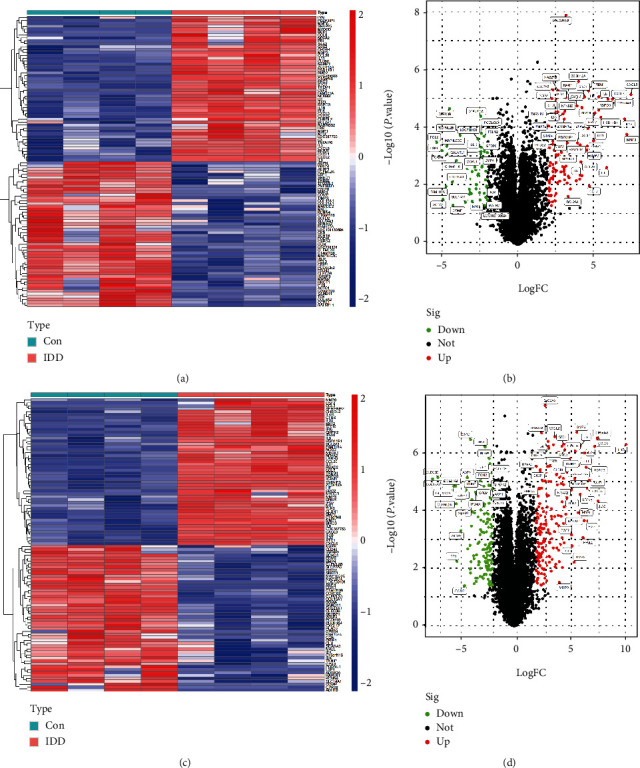
Identification of differentially expressed genes associated with intervertebral disc degeneration (IDD). Heatmap and volcano map of DEGs between IDD patients and healthy control in (a, b) GSE27494 and (c, d) GSE41883. Red represents upregulated genes, green or blue represents downregulated genes, and black represents no significant difference genes.

**Figure 3:**
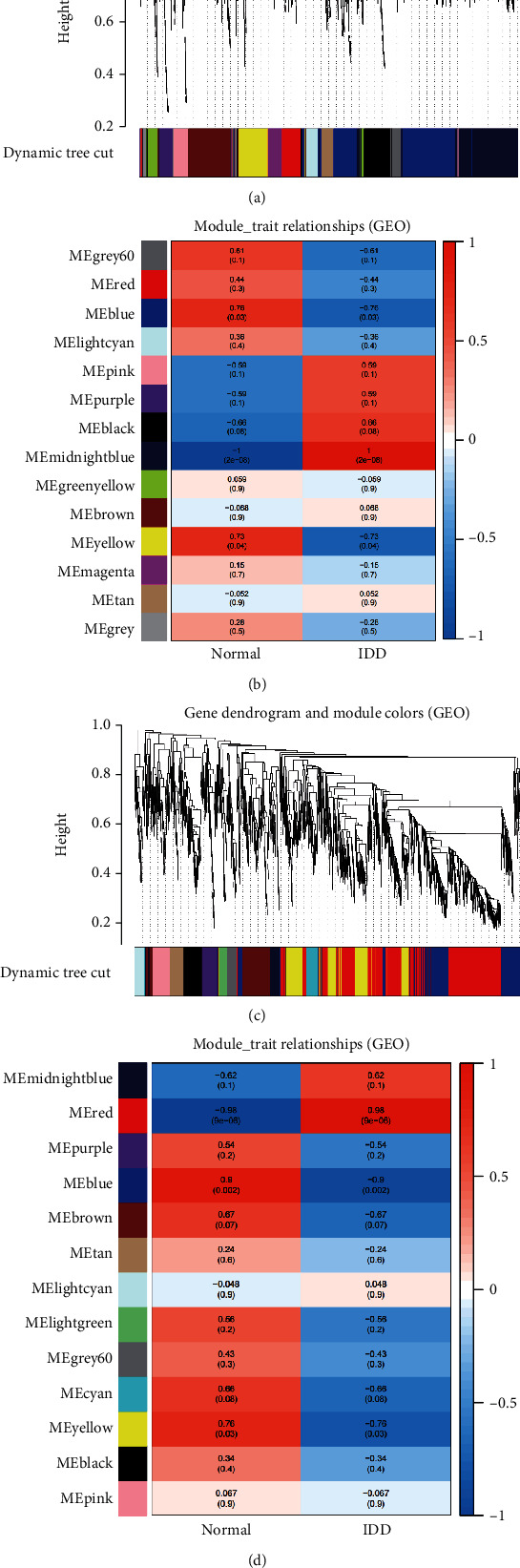
Identification of key modules that relate to IDD via WGCNA. (a, b) The cluster dendrogram of coexpression network modules and module-trait relationships in (a, b) GSE27494 and (c, d) GSE41883. Various colors represent different modules. Each row corresponds to a color module and column corresponds to a clinical trait (IDD and healthy).

**Figure 4:**
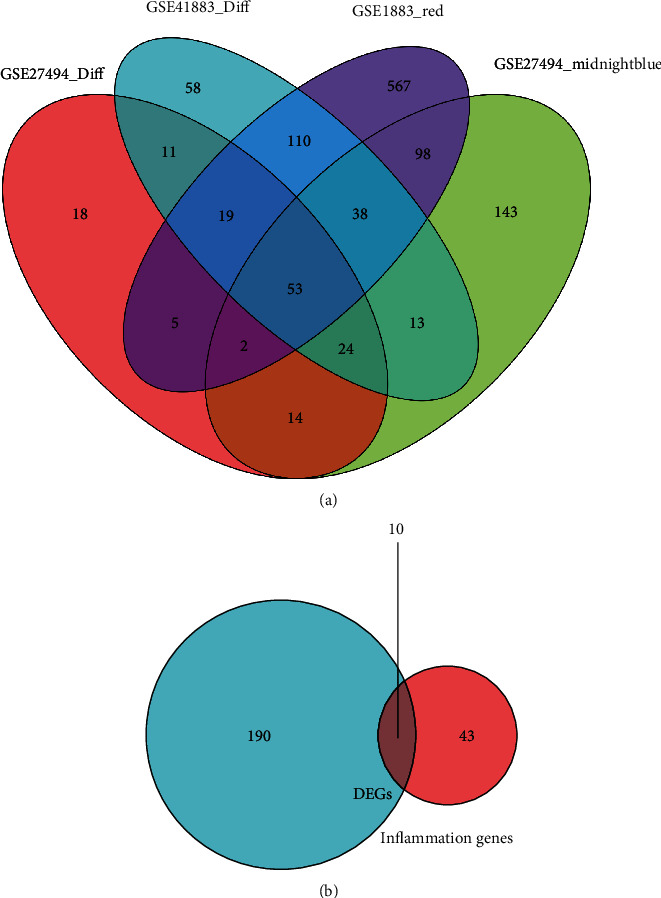
Screening main inflammatory associated genes in IDD. (a) Venn diagram between WGCNA modules and DEGs. (b) The overlapped DEGs and inflammatory associated genes.

**Figure 5:**
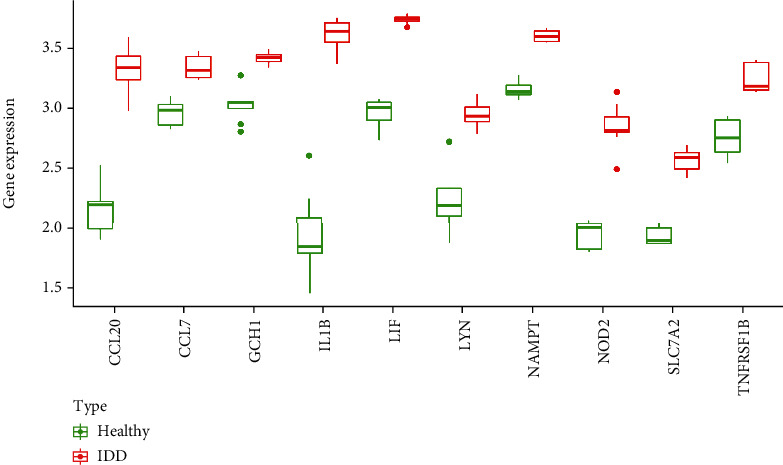
Top 10 differentially expressed inflammatory-related genes in IDD. Boxplots of the expression levels of 10 differentially expressed IRGs in IDD and healthy controls. The green box plots represent the expression in healthy controls, whereas the red box plots represent the expression in IDD.

**Figure 6:**
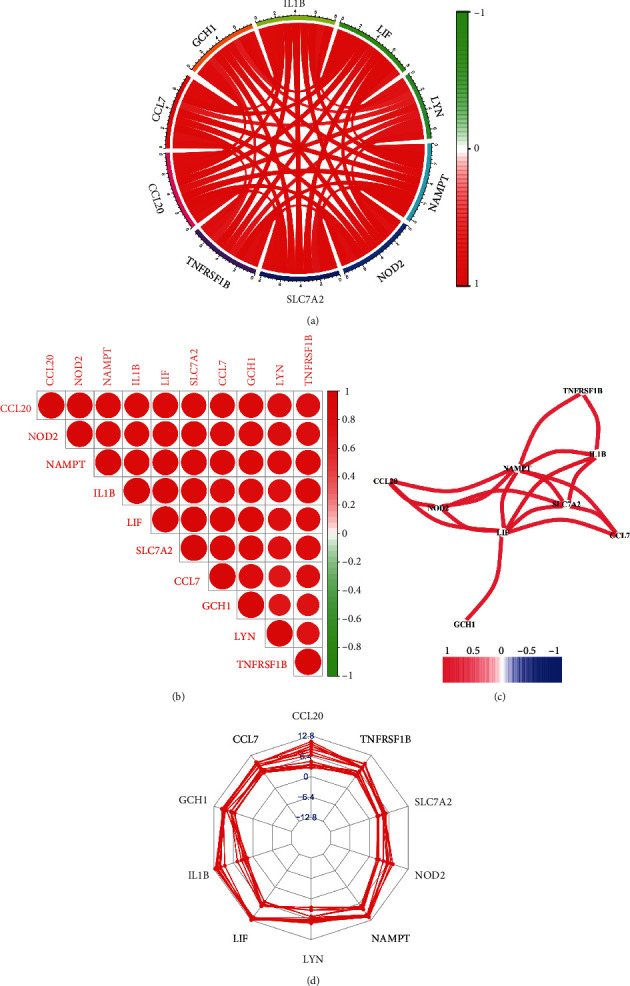
Correlation between differentially expressed IRGs in IDD. (a) Circos plot of differentially expressed IRGs. (b) Correlation plot of differentially expressed IRGs. (c) Correlation network between IRGs. (d) Radar plot of IRGs.

A total of 146 differentially expressed genes were identified in GSE27494, including 50 downregulated and 96 upregulated genes. Meanwhile, a total of 327 differentially expressed genes (including 131 downregulated and 196 upregulated) were obtained in GSE41883. The difference between IDD and normal controls were visualized by heatmap and volcano plot (GSE27494 (Figures 2(a) and 2(b)) and GSE41883 (Figures 2(c) and 2(d))). Then, we used the WGCNA method to explore IDD-associated coexpression modules. A hierarchical clustering tree containing 14 modules in GSE27494 (Figures 3(a) and 3(b)) and 13 modules in GSE41883 (Figures 3(c) and 3(d)) with various colors were constructed. Moreover, the midnight blue module (correlation index: −1, *P* = 2*e* − 08) of GSE27494 and the red module (correlation index: −0.98, *P* = 9*e* − 06) of GSE41883 displayed the highest correlation with IDD. Subsequently, 53 characteristic DEGs were obtained by intersecting differential genes and key module genes of both datasets (Figure 4(a)). Next, 200 inflammatory response-associated genes were downloaded from hallmark gene sets in MSigDB and then overlapped with previously obtained DEGs, and Venn diagrams revealed 10 differentially expressed IRGs (SLC7A2, LIF, NAMPT, IL-1*β*, NOD2, CCL20, CCL7, TNFRSF1B, LYN, and GCH1) (Figure 4(b) and Table 1). Finally, a boxplot revealed that all DE-IRGs were upregulated in IDD group (Figure 5(a)). The relationship between DE-IRGs was further investigated, as shown in Figures 6(a)–6(d).

### 3.2. Functional and Pathway Enrichment Analyses

**Figure 7:**
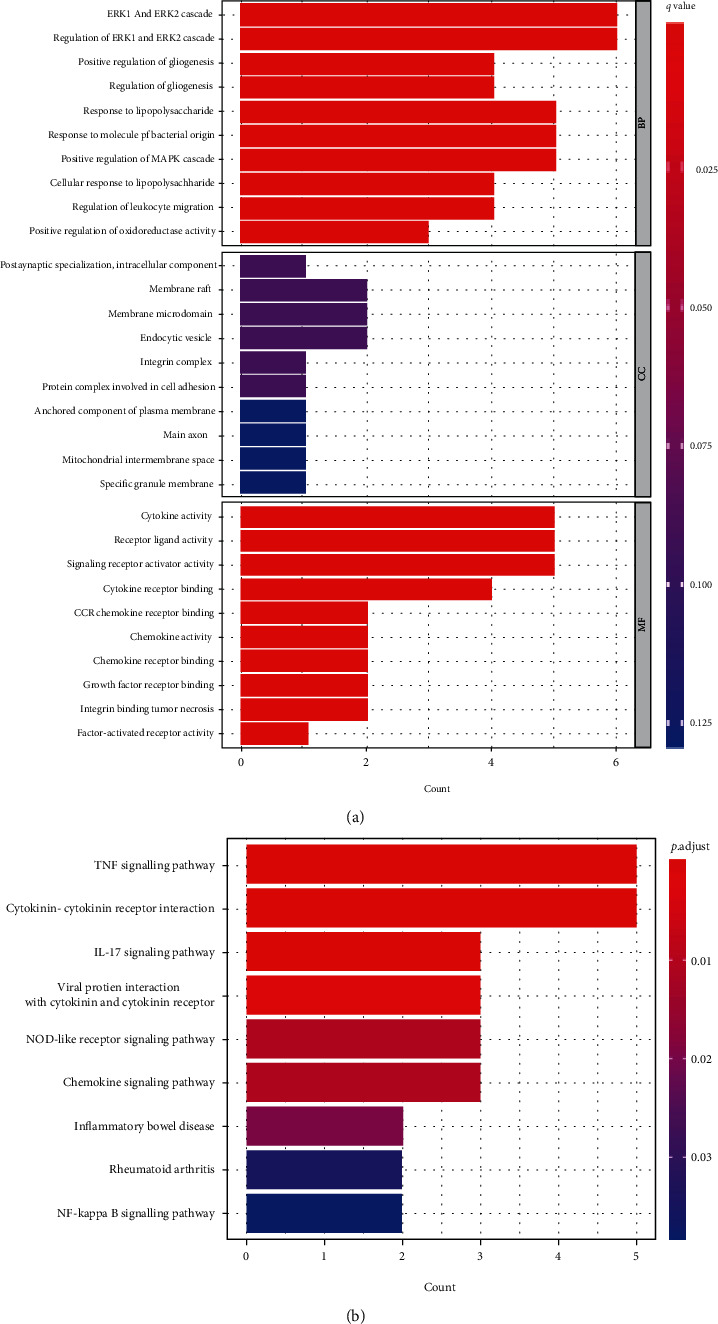
GO and KEGG analyses of DE-IRGs in the pathogenesis of IDD. (a) Significantly enriched GO terms of the DE-IRGs. (b) Significantly enriched KEGG pathways of the DE-IRGs.

Gene Ontology (GO) functional enrichment analysis showed that the identified 10 DE-IRGs were mainly involved in regulation of ERK1 and ERK2 cascade, positive regulation of gliogenesis, response to lipopolysaccharide, response to molecule of bacterial origin, and positive regulation of MAPK cascade (Figure 7(a)). Meanwhile, the results of KEGG enrichment analysis indicated that the selected hub genes played a crucial role in TNF signaling pathway, cytokine-cytokine receptor interaction, IL-17 signaling pathway, viral protein interaction with cytokine, and cytokine receptor and NOD-like receptor signaling pathway (Figure 7(b)).

### 3.3. Screening and Validation of Candidate Signatures

**Figure 8:**
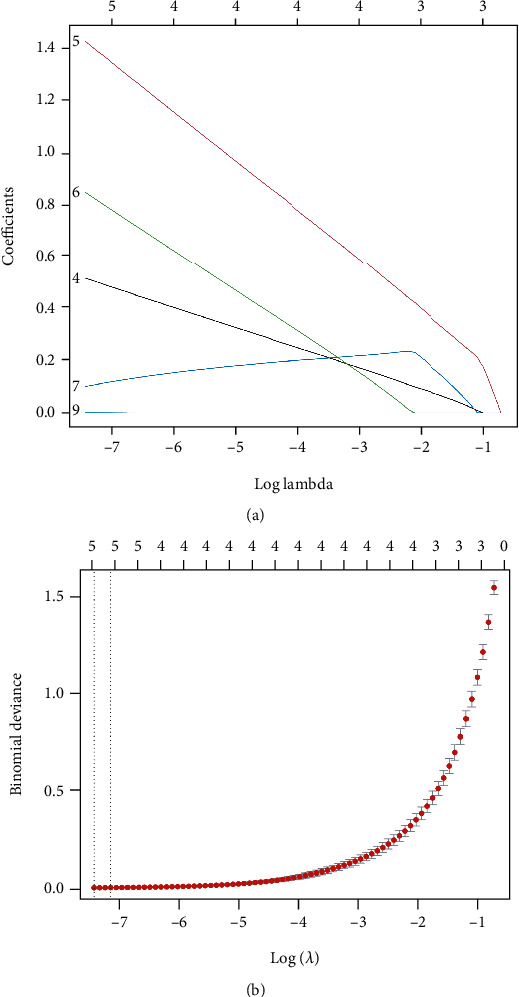
LASSO screen of the hub differentially expressed inflammatory-related genes. (a, b) Least absolute shrinkage and selection operator (LASSO) logistic regression algorithm to screen candidate IRGs.

**Figure 9:**
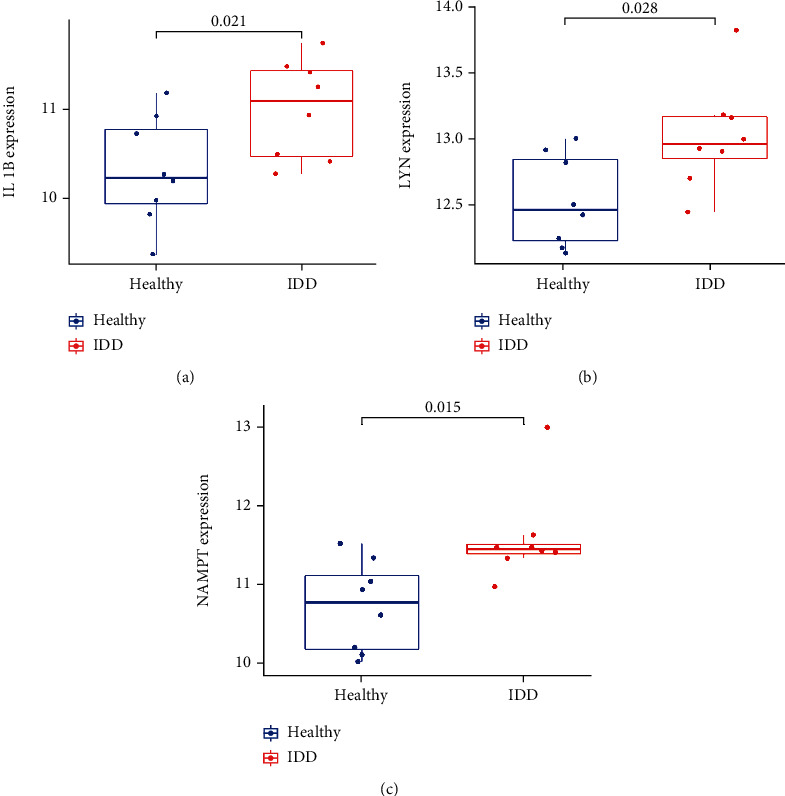
Significant gene expression boxplots of IL-1*β*, LYN, and NAMPT between IDD and healthy controls in GSE150408.

The LASSO regression algorithm was used to reduce overfitting of 10 DE-IRGs and screen the optimal inflammatory related genes associated with IDD. The minimum lambda and 10-fold cross-validation were set to model construction. Finally, five candidate biomarkers (IL-1*β*, LIF, LYN, NAMPT, and SLC7A2) were obtained (Figures 8(a) and 8(b)). To further verify the differentially expression of above genes, we further performed the differential expression analysis in the GSE150408 dataset and found that the expression of IL-1*β*, LYN, and NAMPT was obviously different between IDD and control samples (*P* value < 0.05), as shown in Figures 9(a)–9(c). Hence, IL-1*β*, LYN, and NAMPT were chosen as optical inflammatory associated characteristic genes.

### 3.4. Immune Cell Infiltration

**Figure 10:**
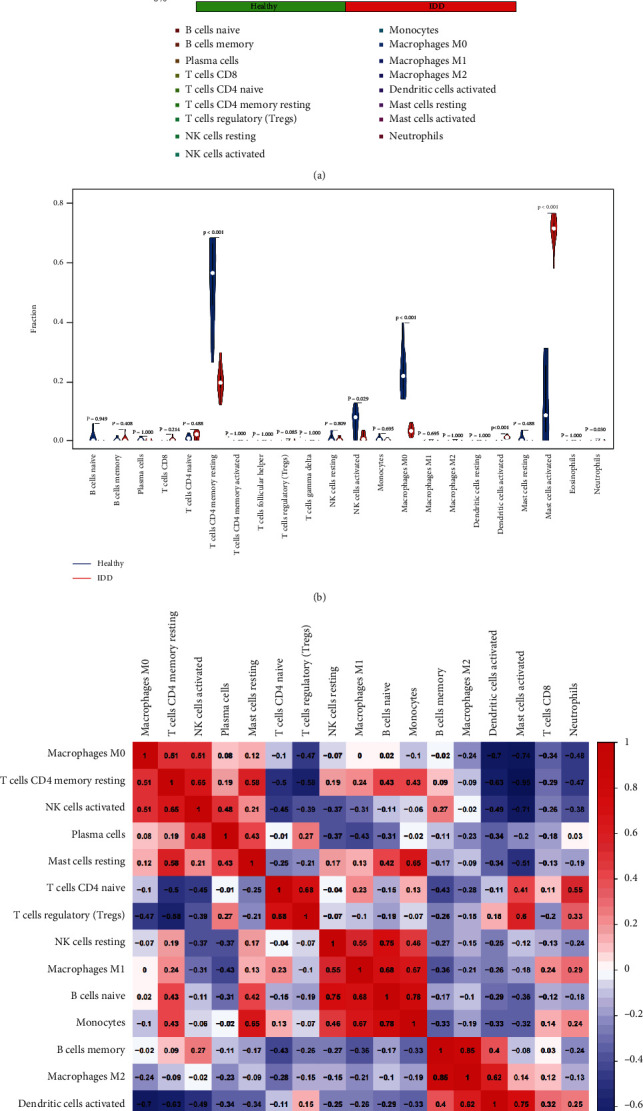
The immune landscape in IDD patients. (a) Bar plot showing the relative proportions of 22 immune cell populations in IDD samples. (b) Violin plot comparing immune cell compositions in the IDD patients and healthy controls. (c) Pearson's correlation analysis of different infiltrating immune cell subpopulations.

The difference of 22 immune cells infiltration between IDD and healthy control was investigated by the CIBERSORT algorithm. The histogram and violin diagram clearly revealed the abundance difference in immune cell infiltration between both groups (Figures 10(a) and 10(b)). The fraction for dendritic cells activated, mast cells activated, and neutrophils in the IDD group was remarkably higher than those in the normal group, while the fraction of some cells was obviously lower than that of the normal group, such as T cells CD4 memory resting, NK cells activated, and macrophage M0 (Figure 10(c)). The above results indicated these differential immune cells might be associated with the immune regulation process of IDD pathogenesis.

### 3.5. Correlation between Three Candidate Biomarkers and Immune-Infiltrated Cells in IDD

**Figure 11:**
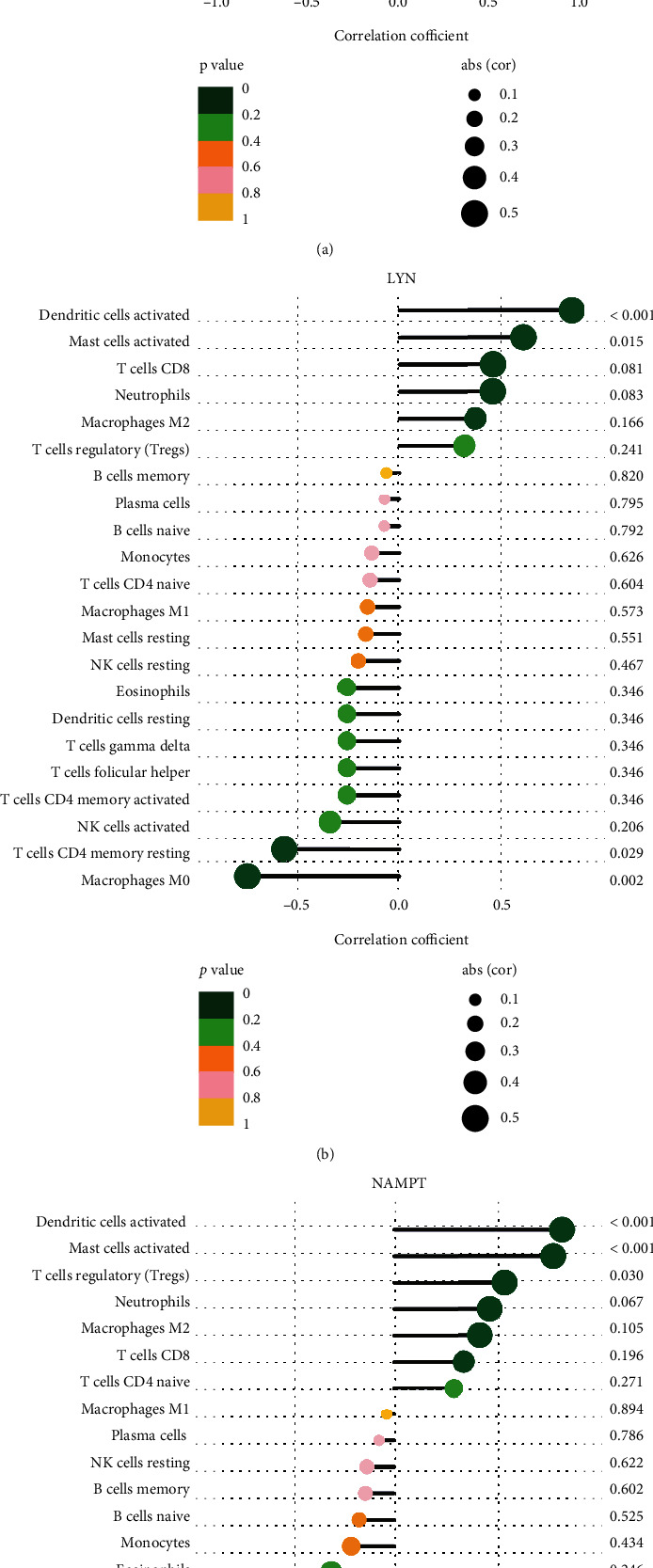
Correlation between immune cells and IL-1*β*, LYN, and NAMPT.

The correlation analysis between optimal DE-IRGs and infiltrating immune cells was further investigated. As shown in Figure 11(a), IL-1*β* displayed a positive correlation with mast cells activated (0.955114313, 8.74*e* − 09), dendritic cells activated (*r* = 0.708, *P* = 0.002), T cells regulatory (Tregs) (*r* = 0.682, *P* = 0.004), neutrophils (*r* = 0.658, *P* = 0.006), and T cell CD4 naïve (*r* = 0.519, *P* = 0.039) and showed a negative correlation with T cells CD4 memory resting (*r* = −0.96, *P* < 0.01), macrophage M0 (*r* = −0.72, *P* = 0.002), NK cells activated (*r* = −0.672, *P* = 0.004), and mast cells resting (*r* = −0.57, *P* = 0.021). As shown in Figure 11(b), LYN showed a positive correlation with mast cells activated (*r* = 0.593, *P* = 0.015) and dendritic cells activated (*r* = 0.823, *P* < 0.01) and showed a negative correlation with macrophage M0 (*r* = −0.729, *P* = 0.002), T cell CD4 memory resting (*r* = −0.553, *P* = 0.029), and NK cells activated (*r* = −0.334, *P* = 0.206). As shown in Figure 11(c), NAMPT showed a positive correlation with mast cells activated (*r* = 0.78, *P* < 0.01) and dendritic cells activated (*r* = 0.821, *P* < 0.01) and indicated a negative correlation with T cell CD4 memory resting (*r* = −0.776, *P* < 0.01) and macrophage M0 (*r* = −0.626, *P* = 0.011). These results suggested that the interplay between inflammatory associated genes and immune cells played a critical role in the progression of IDD.

## 4. Discussion

Intervertebral disc degeneration (IDD) is a major cause of low back pain, which leads to high social and economic cost [9]. Low back pain has been identified as one of the most common reasons for seeking medical care [10]. With an increasing prevalence in the aging population, there is an urgent need to elucidate the etiology and find out the best therapy of IDD. To date, the diagnosis of IDD largely relies on symptoms and imaging, which hinders early diagnosis and timely treatment. Despite years of efforts and attempts, the exact mechanisms of IDD remain unclear, and effective treatments are still lacking. Although some biomarkers have been identified in previous studies, there is no focus on the comprehensive investigation of immune cells and inflammatory genes in IDD. Thus, we aimed to explore potential inflammatory associated signature of IDD and further investigate their relationship with immune infiltration based on comprehensive analysis.

In the present study, we used the WGCNA and the CIBERSROT algorithm to screen characteristic inflammatory associated genes and immune cells related to IDD development. Firstly, we obtained 10 inflammatory-related genes by overlapping genes from differentially expressed analysis and key modules of the WGCNA. Next, GO and KEGG pathway analyses found that these genes are mainly involved in TNF, IL-17, NOD-like receptor, and NF-kappa B signaling pathway, indicating that inflammation response is an important pathological process of IDD. Moreover, IL-1*β*, LYN, and NAMPT were identified as hub candidate genes by the LASSO regression and external validation. Finally, we discovered different immune cells infiltration landscape between IDD patients and healthy control and found that IL-1*β*, LYN, and NAMPT were closely implicated with immune response. To the best of our knowledge, studies of IRGs and immune response related to IDD are limited, and our study may provide new insights into the pathogenesis of IDD by exploring the cross talk between hub inflammatory genes and immune cells.

To date, a series of studies have demonstrated that proinflammatory molecules are potent factors in initiation and progression of IDD [11, 12]. Many important inflammatory biomarkers have been investigated and have the potential to guide diagnosis and therapeutics [13]. IL-1*β* is one of the most important proinflammatory cytokine which promoted ECM degradation, oxidative stress, and NLRP3 inflammasome activation in NP cells, and drugs targeting IL-1*β* play a protective role against inflammation injury [14–16]. Nampt is a rate-limiting enzyme for the NAD+ remedial synthetic pathway in mammalian cells, which plays a critical role in regulating cellular senescence [17]. However, the biological effect of Nampt on IDD remains controversial. Some studies showed that Nampt stimulated the activation of NLRP3 inflammasome and promoted the progression of IDD [18]. Nampt inhibitor, for example, APO866, prevented ECM degradation and NP cells apoptosis, indicating that Nampt is harmful for NP cells via inflammation activation [19]. On the other side, some studies supported that Nampt is beneficial for IDD. Sun et al. found that delivering exogenous Nampt enhanced NAD^+^ biosynthesis in senescent NP cells and delayed the development of IDD in rats [20]. Shi et al. reported that resveratrol activated autophagy and attenuated IDD via activation of the Nampt/NAD+/SIRT1 pathway [21]. Hence, the influence of Nampt on inflammatory response and senescence needs further investigation. Of the significantly enriched pathways in the KEGG pathway analysis, the NOD-like receptor signaling pathway was of interest as it played an important role in inflammation response based on literatures. The activation of NOD-like receptor protein 3 (NLRP3) inflammasome has been shown to promote inflammation by release of potent proinflammatory cytokines interleukin- (IL-) 1*β* and IL-18. In recent years, with further exploration of inflammasomes, growing evidence has demonstrated that inflammasomes are associated with the occurrence and progression of IDD [22]. NLRP3 inflammasome activation was significantly increased in degenerated disc and IDD model, and drugs targeting NLRP3, such as cortistain [23], melatonin [24], and mesenchymal stem cell- (MSC-) derived exosomes [25], ameliorated intervertebral disc degeneration via repressing NLRP3 activation. As mentioned above, inflammation response exacerbates the severity of IDD. On the other hand, anti-inflammation therapy could alleviate IVD degeneration. For example, IL-10 was an important anti-inflammatory mediator, and exogenous IL-10 treatment delayed IVD degeneration via inhibiting p38 MAPK activation and inflammation response [26].

Immune response is an important driver of inflammation, and various immune cells play different roles in the process of IDD. Intervertebral disc has been widely accepted as an immune privilege organ because of its avascular structure. However, the rupture of outer annulus fibrosus results in exposure of inner nucleus pulposus to circulation and consequently triggers an autoimmune reaction. The infiltration of immune cells, such as neutrophils, T cells, and macrophages might release a large amount of proinflammatory molecules and promoted inflammation cascade within the disc. Wang et al. showed the immune infiltration landscape varied significantly between LDH and healthy control, and both Tregs and macrophages were implicated with IDD development [7]. Macrophage has been identified as a key immune player in the process of IDD. Significant interaction between macrophages and progenitor NP cells via MIF (macrophage migration inhibitory factor) and NF-*κ*B signaling pathways was found during the progression of IDD via single-cell RNA sequencing [27]. To be mentioned, the function of macrophage is influenced by both phenotypes and microenvironment. It was reported that after cocultured with IL-1*β* and IVD-conditioned medium, macrophages prevented IVD ECM remodeling and decreased aggrecan and collagen II gene expression in the presence of IL-1*β* [28]. The cross talk between macrophage and IVD in degenerated disc tended to polarize macrophages toward a more proinflammatory state, which accelerated IVD degeneration as a return. A recent study showed that magnoflorine alleviated M_1_ macrophage-induced IDD by the inhibition of NLRP3 inflammasome activation [29]. On the other hand, the M_2_ macrophage played an anti-inflammatory effect and increased NP cell proliferation and ECM synthesis under TNF-*α* stimulation [30]. Nowadays, increasing evidence support that M_1_ polarization shows a proinflammatory effect while M_2_ state plays an anti-inflammation and remodeling effect in response to injury [31–33]. Hence, future macrophage-anchored therapeutics is promising in the management of IDD. Moreover, many other types of immune cells, including neutrophils and T cells, have attracted a lot of research attention and the progress of interaction between immune cells and inflammatory response is expected to become a breakthrough in the treatment of IDD.

Notably, there are still few studies on the interaction of inflammation related genes and immune infiltration in the pathogenesis of IDD. Our rigorous bioinformatic analysis provided reliable inflammatory genetic biomarkers in the IDD model and paved the way for future therapeutic strategies research for IDD by targeting inflammatory genes and immune modulation. Nevertheless, there were certain limitations in our study. First, this study was completely based on public datasets with small sample size, which might result in biased interpretation. Second, in vivo and in vitro experiments for validation are lacking. Hence, further in-depth biological experiments on immune infiltration and inflammatory response are required.

## 5. Conclusion

In conclusion, the present study revealed that the expression level of inflammatory associated genes (IRGs) and immune infiltration landscape significantly differed between IDD patients and healthy control. Additionally, based on this comprehensive bioinformatic analysis, we identified hub IRGs, critical regulatory pathways and immune infiltration characteristics of IDD. Moreover, the correlation between hub IRGs and immune cells was analyzed. In all, these findings may extend our knowledge concerning inflammation response and immune modulation in IDD patients.

## Figures and Tables

**Table 1:** Top 10 inflammatory response-associated genes in IDD.

Gene	logFC	AveExpr	*t*	*P* value	Adj. *P* value	*B*
CCL20	5.532308225	6.259162738	10.74688389	2.19*e* − 08	6.75*e* − 06	9.69840814
CCL7	2.316937	7.937576375	7.569315372	1.84*e* − 06	0.000129624	5.296735863
GCH1	2.495734375	8.387334613	8.301695527	5.98*e* − 07	6.30*e* − 05	6.422200711
IL1B	8.303783088	7.093097344	16.30224347	7.29*e* − 11	8.45*e* − 08	15.09109203
LIF	5.524410525	9.511424375	20.88386735	2.16*e* − 12	7.80*e* − 09	18.1460731
LYN	2.870808	5.271320438	6.57260378	9.44*e* − 06	0.000413442	3.645774035
NAMPT	3.215158813	9.482475856	16.14269113	8.37*e* − 11	9.17*e* − 08	14.9660275
NOD2	3.35102225	4.538360288	9.85462812	6.82*e* − 08	1.40*e* − 05	8.582475124
SLC7A2	2.1113183	3.863241175	14.32074519	4.46*e* − 10	3.14*e* − 07	13.42836733
TNFRSF1B	2.731989775	7.10356355	7.253318035	3.04*e* − 06	0.000188559	4.788480163

## Data Availability

The following information was supplied regarding data availability: The raw data can be found at https://www.ncbi.nlm.nih.gov/geo/query/acc.cgi?acc=GSE27494, https://www.ncbi.nlm.nih.gov/geo/query/acc.cgi?acc=GSE41883, and https://www.ncbi.nlm.nih.gov/geo/query/acc.cgi?acc=GSE150408.

## Abbreviations

LBP: Low back pain
IVD: Intervertebral disc
IDD: Intervertebral disc degeneration
AF: Annulus fibrosus
NP: Nucleus pulposus
CEP: Cartilage endplate
ECM: Extracellular matrix
MMP: Matrix metalloproteinase
DEGs: Differentially expressed genes
BP: Biological process
CC: Cellular component
MF: Molecular function
KEGG: Kyoto Encyclopedia of Genes and Genomes.
